# A Toxin-Binding Alkaline Phosphatase Fragment Synergizes Bt Toxin Cry1Ac against Susceptible and Resistant *Helicoverpa armigera*

**DOI:** 10.1371/journal.pone.0126288

**Published:** 2015-04-17

**Authors:** Wenbo Chen, Chenxi Liu, Yutao Xiao, Dandan Zhang, Yongdong Zhang, Xianchun Li, Bruce E. Tabashnik, Kongming Wu

**Affiliations:** 1 The State Key Laboratory for Biology of Plant Disease and Insect Pests, Institute of Plant Protection, Chinese Academy of Agricultural Sciences, West Yuanmingyuan Road, Beijing, 100193, China; 2 Department of Entomology, University of Arizona, Tucson, AZ, 85721, United States of America; University of Tennessee, UNITED STATES

## Abstract

Evolution of resistance by insects threatens the continued success of pest control using insecticidal crystal (Cry) proteins from the bacterium *Bacillus thuringiensis* (Bt) in sprays and transgenic plants. In this study, laboratory selection with Cry1Ac yielded five strains of cotton bollworm, *Helicoverpa armigera*, with resistance ratios at the median lethal concentration (LC_50_) of activated Cry1Ac ranging from 22 to 1700. Reduced activity and reduced transcription of an alkaline phosphatase protein that binds Cry1Ac was associated with resistance to Cry1Ac in the four most resistant strains. A Cry1Ac-binding fragment of alkaline phosphatase from *H*. *armigera* (HaALP1f) was not toxic by itself, but it increased mortality caused by Cry1Ac in a susceptible strain and in all five resistant strains. Although synergism of Bt toxins against susceptible insects by toxin-binding fragments of cadherin and aminopeptidase N has been reported previously, the results here provide the first evidence of synergism of a Bt toxin by a toxin-binding fragment of alkaline phosphatase. The results here also provide the first evidence of synergism of a Bt toxin by any toxin-binding peptide against resistant insects.

## Introduction

The insecticidal proteins of *Bacillus thuringiensis* (Bt) kill some major insect pests, but are harmless to vertebrates and most other organisms [1–3]. In 2013, farmers planted genetically modified corn and cotton producing Bt toxins on 76 million hectares worldwide [4]. Widespread use of Bt toxins in sprays and transgenic crops has caused field-evolved resistance to Bt toxins in some pests, which entails a genetically based decrease in susceptibility [5, 6]. Some degree of field-evolved resistance has been reported in two pest species exposed to Bt sprays and in nine pest species exposed to Bt crops [7–21].

The most widely used Bt proteins are crystalline (Cry) toxins, particularly Cry1Ab in Bt corn and Cry1Ac in Bt cotton that kill lepidopteran larvae [2, 6]. A key step in the mode of action of Cry1A toxins is binding of activated toxin to midgut membrane proteins [3]. Mutations that interfere with this step can confer resistance to Bt toxins [3, 22–24]. Insect midgut proteins that bind Cry toxins and are considered receptors for these toxins include cadherin, aminopeptidase N (APN), and alkaline phosphatase (ALP) [3, 24]. Disruption or reduced expression of the genes encoding these toxin-binding proteins is a common mechanism of resistance to Bt toxins in Lepidoptera [3, 22–31].

In many cases, toxin-binding fragments of cadherin or APN are not toxic alone, but they interact with Bt toxins either as synergists that increase mortality [32–46] or as antagonists that decrease mortality [45, 47–51]. Previous work, however, has not examined if ALP fragments synergize or antagonize Bt toxins. Moreover, many researchers have proposed that synergistic fragments of Bt toxin receptors might be useful for delaying or countering resistance, yet previous work has tested these fragments only against susceptible strains of pests [32–51].

Here we tested for synergism of Cry1Ac by an ALP fragment in susceptible and resistant strains of the cotton bollworm, *Helicoverpa armigera*, the most serious pest of cotton in China [52]. Bt cotton producing Cry1Ac was introduced to China in 1997 and has achieved great success against *H*. *armigera* [53, 54], yet monitoring has provided an early warning of field-evolved resistance to Cry1Ac in this pest [16, 30, 31, 55]. Previous work showed that Cry1Ac binds to two ALPs from *H*. *armigera* called HaALP1 and HaALP2 [56]. We analyzed the relationship between ALP and resistance to Cry1Ac in five laboratory-selected strains of *H*. *armigera* derived from a common susceptible strain from China. The results show that reduced activity and transcription of ALP were associated with resistance to Cry1Ac in the four most resistant strains studied here. We also discovered that a fragment of HaALP1, which we name HaALP1f, synergized Cry1Ac against larvae from a susceptible strain and all five resistant strains.

## Results

### Resistance associated with activity and transcription of ALP, but not with activity of APN

**Fig 1:**
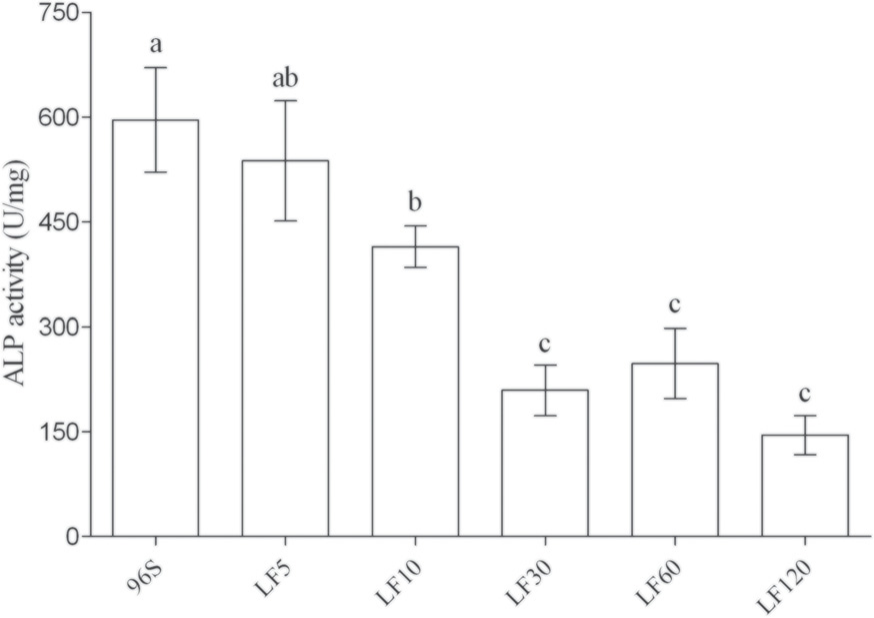
ALP activity in BBMV from susceptible and resistant *H*. *armigera* larvae. Different letters above the error bars indicate significant differences between means (P < 0.05).

**Fig 2:**
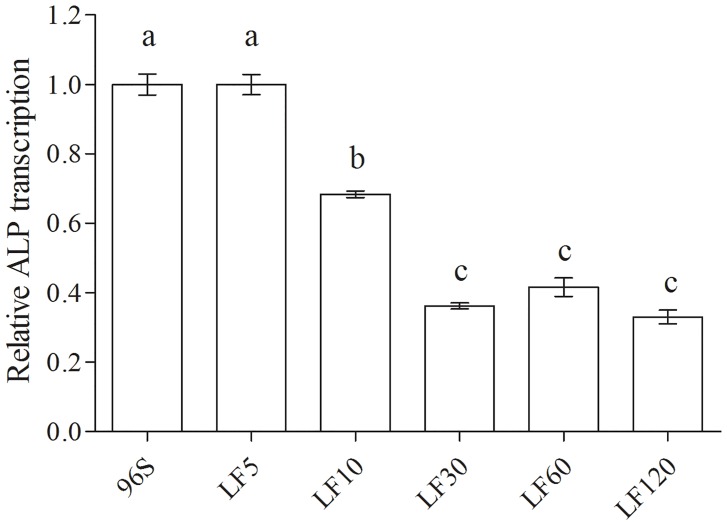
Relative ALP transcription detected by qRT-PCR in susceptible and resistant *H*. *armigera* larvae. Different letters above the error bars indicate significant differences between means (P<0.05).

**Fig 3:**
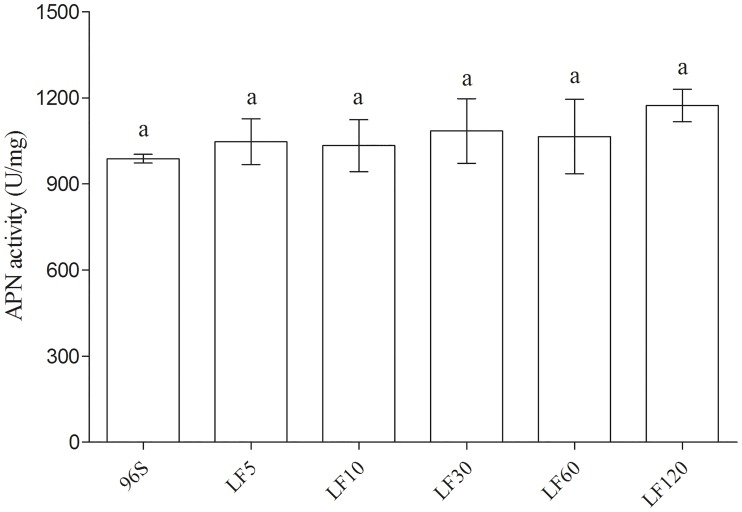
APN activity in BBMV from susceptible and resistant *H*. *armigera* larvae. The same letter (a) above the standard error bars indicates no significant differences in mean APN activity among strains (P > 0.05).

Relative to a susceptible strain (96S), laboratory selection yielded resistance ratios based on the median lethal concentration (LC_50_) of activated Cry1Ac ranging from 22 to 1700 in five strains of *H*. *armigera* (Table 1). Reduced ALP activity and transcription were associated with resistance to Cry1Ac in the four most resistant strains (LF10, LF30, LF60, and LF120), but not in the least resistant of the five selected strains (LF5) (Figs 1 and 2). ALP activity and transcription did not differ significantly between LF5 and the susceptible strain, but LF10, LF30, LF60 and LF120 had significantly lower ALP activity and transcription than the susceptible strain (Figs 1 and 2). As expected, ALP activity and ALP transcription were positively associated across all observations for the six susceptible and resistant strains of *H*. *armigera* (linear regression, F_1, 34_ = 58.6, R^2^ = 0.63, P < 0.0001). The resistance ratio for Cry1Ac activated toxin (log-transformed) was negatively associated with mean ALP activity across the six susceptible and resistant strains (linear regression, F_1, 4_ = 35.1, R^2^ = 0.90, P = 0.004). APN activity did not vary significantly among the six strains (Fig 3).

**Table 1:** Susceptibility of *H*. *armigera* strains to Cry1Ac.

Strain	LC_50_ (95% fiducial limits)(μg Cry1Ac per ml diet)	Resistanceratio*
96S (susceptible)	0.013 (0.007–0.021)	1.0
LF5 (resistant)	0.286 (0.18–0.42)	22
LF10 (resistant)	0.624 (0.45–0.86)	48
LF30 (resistant)	3.60 (2.1–5.5)	280
LF60 (resistant)	9.15 (5.8–13)	700
LF120 (resistant)	22.1 (16–31)	1700

* LC_50_ of each strain divided by the LC_50_ of the susceptible strain 96S.

### Production and characterization of *H*. *armigera* ALP fragment (HaALP1f)

**Fig 4:**
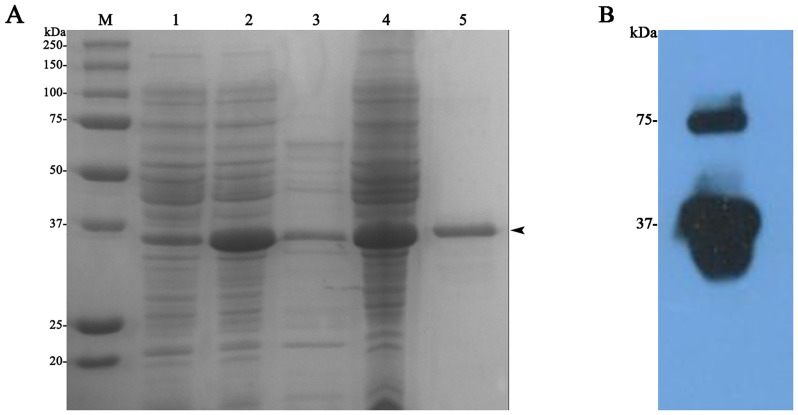
Detection of HaALP1f expressed in *E*. *coli*. (A) SDS-PAGE separation of the protein extracts from HaALP1f-expressing *E*. *coli* cells. M: Molecular weight markers; Lane 1: total extract from non-induced HaALP1f-expressing *E*. *coli* cells; Lane 2: total extract from IPTG-induced HaALP1f-expressing *E*. *coli* cells; Lane 3: the supernatant (from 20 min centrifugation at 25,000×g) of total extract from IPTG-induced HaALP1f-expressing *E*. *coli* cells; Lane 4: The pelleted inclusion body (from 20 min centrifugation at 25,000×g) of total extract from IPTG-induced HaALP1f-expressing *E*. *coli* cells; Lane 5: Purified HaALP1f from the pelleted inclusion body from IPTG-induced HaALP1f-expressing *E*. *coli* cells by using Ni-affinity column. (B) Detection of purified HaALP1f by Western blot.

Using PCR amplification, we cloned a previously described 780 bp cDNA fragment of the gene from *H*. *armigera* encoding HaALP1 [56]. The peptide encoded by this cDNA fragment, referred to here as HaALP1f, is predicted to have 260 amino acid residues (^192^A-T^451^) and a molecular weight of 30 kDa. SDS-PAGE analysis of the total extracts of *Escherichia coli* cells transformed with the his-tagged HaALP1f-pET28a+ construct revealed a protein band of the expected size (about 35 kDa, indicated by a black arrowhead in Fig 4A. This band was not only the strongest protein band, but also the only inducible band by IPTG (compared lane 1 and 2 in Fig 4A). This band was present in the supernatant (lane 3 in Fig 4A) and the pelleted inclusion bodies (lane 4 in Fig 4A). After purification with Ni-affinity column that captures his-tagged proteins, the 35–37 kDa band was the only visible band on the gel (lane 5 in Fig 4A). Western blot hybridization of the Ni column-purified proteins from IPTG-induced HaALP1f-expressing *E*. *coli* cells with the anti-his antibody showed that the visible 35–37 kDa band was strongly hybridized with the anti-his antibody, confirming that this band represented the his-tagged HaALP1f (Fig 4B). Western blot also revealed a weak positive band of around 74 kD (Fig 4B), suggesting that a small portion of the heterologously expressed HaALP1f existed as a homodimmer.

### Cry1Ac binding to HaALP1f

**Fig 5:**
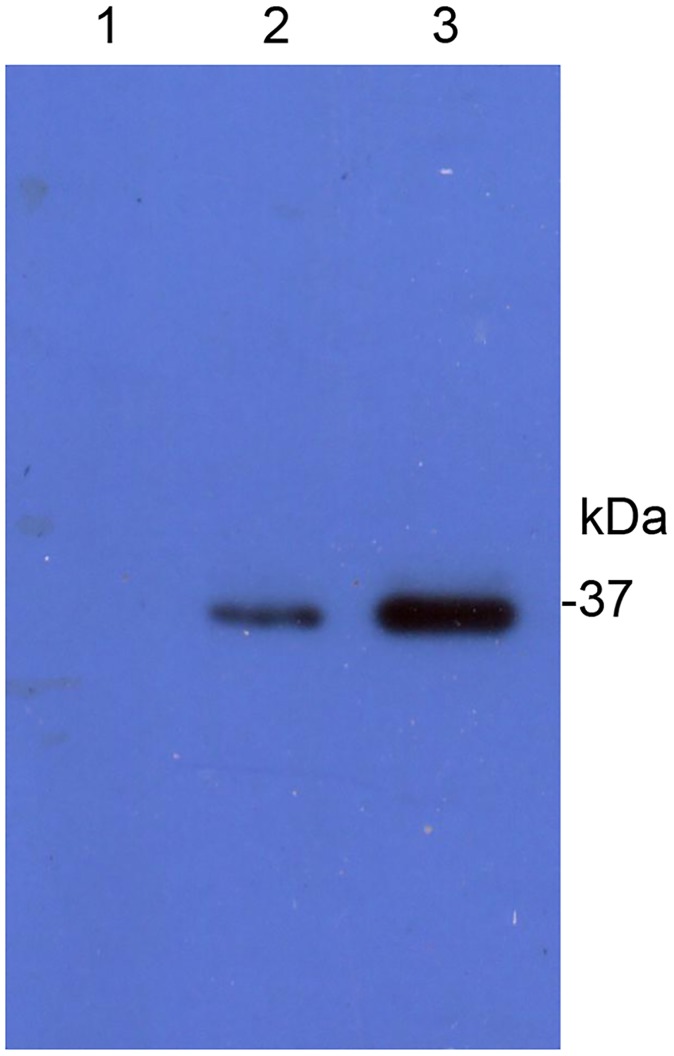
Binding of Cry1Ac to purified ALP fragment (HaALP1f) in ligand blot. The proteins transferred to a PVDF membrane were probed with activated Cry1Ac toxin and detected by a polyclonal anti-Cry1Ac antibody. Lane 1: lysates of *E*.*coli* cells transformed with the empty pET28a+ vector; Lane 2: lysates of *E*. *coli* cells transformed with HaALP1f-pET28a+ construct, Lane 3: purified HaALP1f from *E*. *coli* cells transformed with HaALP1f-pET28a+ construct.

**Fig 6:**
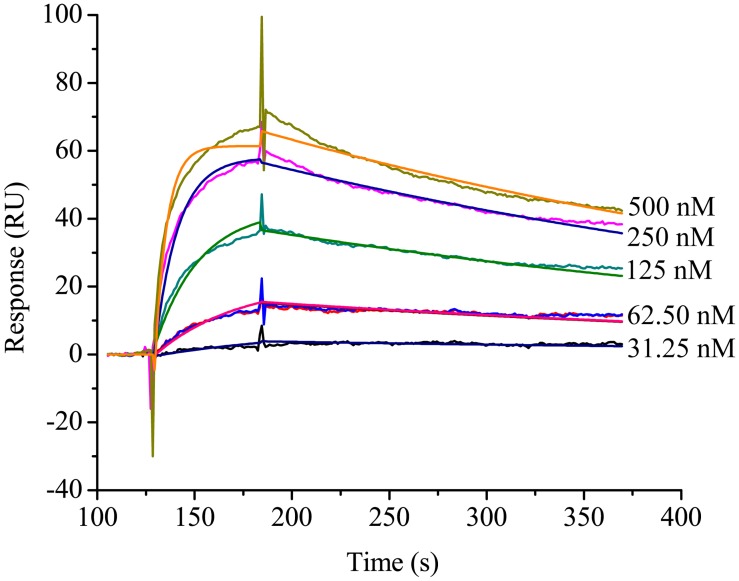
The affinities of binding of Cry1Ac to HaALP1f. Experimental curves (jagged line) are shown overlaid with fitted curves (smooth line) obtained with the 1:1 Langmuir binding model. The overlaid BIAcore response curves are shown for HaALP1f injections at 31.25, 62.5, 125, 250, and 500 nM.

Ligand blot analysis revealed that the lysates of *E*. *coli* cells expressed with his-tagged HaALP1f had a protein of the same size with the his-tagged HaALP1f (35–37 kDa) that bound to the activated Cry1Ac (lane 2 in Fig 5). And the intensity of the Cry1Ac-binding protein band was much stronger in the Ni column-purified proteins from IPTG-induced ALP-expressing *E*. *coli* cells (lane 3 in Fig 5) than in the crude lysates of *E*. *coli* cells expressed with his-tagged HaALP1f. By contrast, the lysates of control *E*. *coli* cells transformed with the empty pET28a+ vector did not have a protein capable of binding to the activated Cry1Ac (Lane 1 in Fig 5). In addition, overlay plot of the surface plasmon resonance (SPR) sensorgrams between different concentrations of the purified HaALP1f and immobilized activated Cry1Ac also showed HaALP1f-activated Cry1Ac binding interaction (Fig 6). A 1:1 binding stoichiometry produced the following apparent rate constants of the bimolecular interaction: k_a_ = 3.27× 10^5^ M^-1^s^-1^ and k_d_ = 2.48 × 10^-3^ s^-1^, K_D_ = 7.58 nM.

### HaALP1f synergizes Cry1Ac against susceptible and resistant strains

**Fig 7:**
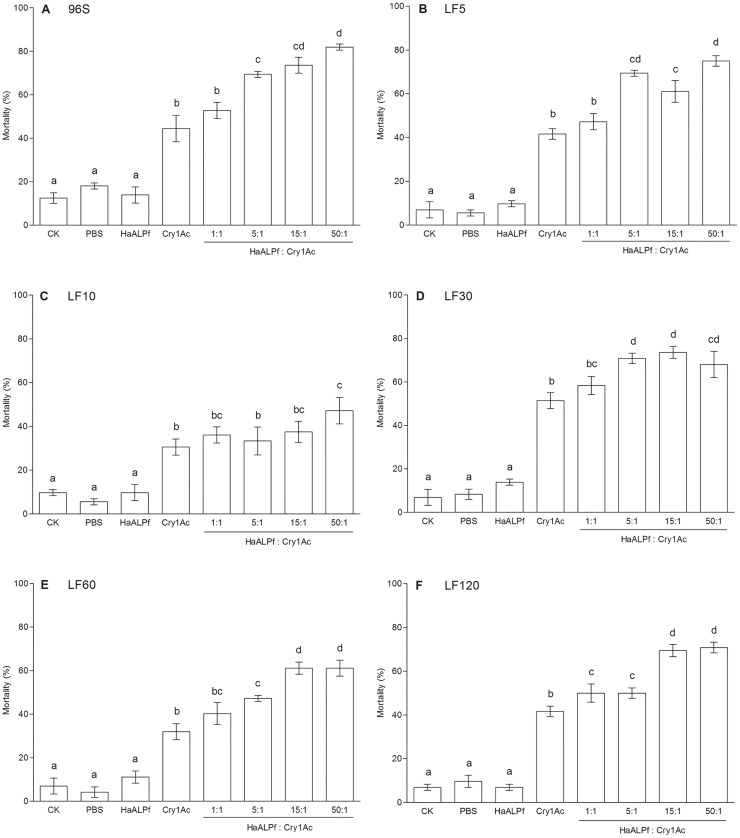
Synergism of Cry1Ac activated toxin by HaALP1f against susceptible and resistant strains of *H*. *armigera*. CK: untreated diet, PBS: diet treated with PBS buffer, HaALP1f: diet treated with HaALP1f alone, and Cry1Ac: diet treated with Cry1Ac activated toxin alone. The ratios above HaALP1f:Cry1Ac indicate the ratio of HaALP1f to Cry1Ac activated toxin by weight. Different letters above the error bars indicate significant differences between means (P < 0.05).

**Fig 8:**
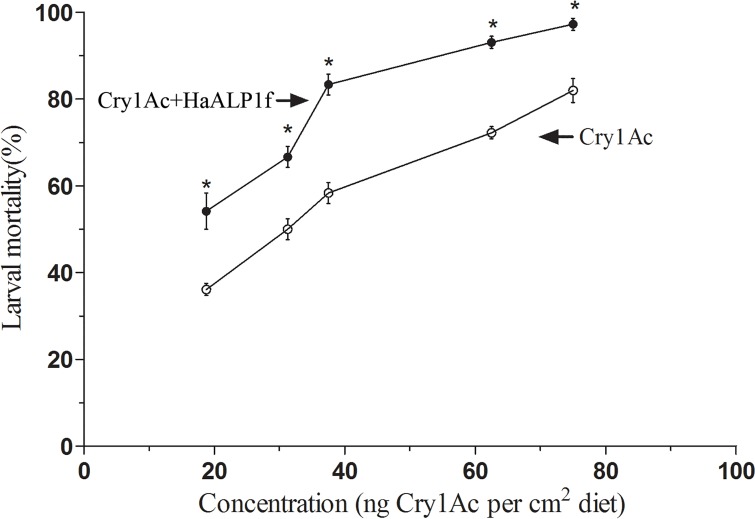
Effect of HaALP1f on toxicity of Cry1Ac activated toxin to a susceptible strain (96S) of *H*. *armigera*. The ratio of HaALP1f:Cry1Ac was 15:1 by weight. Asterisks denote significantly higher mortality with a mixture of HaALP1f and Cry1Ac than with Cry1Ac alone (P < 0.05) at a particular concentration of Cry1Ac.

We tested for synergism in a series of bioassays that examined mortality caused by Cry1Ac activated toxin alone, HaALP1f alone, and combinations of various concentrations of Cry1Ac activated toxin and HaALP1f. For each of the six susceptible and resistant strains of *H*. *armigera*, one or more combinations of HaALP1f and Cry1Ac caused significantly higher mortality than Cry1Ac alone (Figs 7 and 8). For each of the six strains, HaALP1f was not toxic by itself, as indicated by the lack of significant difference in mortality between HaALP1f in PBS buffer alone and the control with only the PBS buffer (6 pairwise comparisons, P > 0.05 in each comparison, Fig 7). In addition, with the data from all six strains pooled, mortality did not differ significantly between the PBS buffer with HaALP1f (mean = 10.9%, 95% confidence interval = 8.6 to 13.2) and the PBS buffer without HaALP1f (mean = 8.6%, 95% confidence interval = 5.8 to 11.4%; t-test, t = 1.36, df = 34, P = 0.18). Therefore, the significantly increased mortality seen with the combination of HaALP1f and Cry1Ac was caused by synergism, rather than independent toxicity of HaALP1f.

For each strain, we tested a fixed concentration of Cry1Ac that by itself caused 23 to 48% mortality (mean = 35%), with the concentration ranging from 37 ng/cm^2^ for the susceptible strain to 1500 ng/cm^2^ for the most resistant strain (LF120) (Fig 7). For each strain, we tested HaALP1f in combination with the fixed concentration of Cry1Ac in ratios of HaALP1f:Cry1Ac of 1:1, 5:1, 15:1 or 50:1 by weight. For all six strains, the 50:1 ratio of HaALP1f:Cry1Ac significantly increased mortality compared with Cry1Ac alone, with a mean increase in mortality of 26% (range = 6.9 to 38%) (Fig 7).

For all strains except LF10, ratios of 15:1 and 5:1 HaALP1f:Cry1Ac also significantly increased mortality compared with Cry1Ac alone (Fig 7). At the 1:1 ratio of HaALP1f:Cry1Ac, mortality with the mixture was significantly higher than with Cry1Ac alone only for the most resistant strain (LF120) and the mean increase in mortality for all six strains was 7.2% (range = 5.6 to 8.3%) (Fig 7). For all strains, the increase in mortality was significantly higher for HaALP1f:Cry1Ac at 50:1 compared with 1:1 (U test, P = 0.02). The increase in mortality caused by adding HaALP1f to Cry1Ac did not differ significantly between the susceptible strain (mean = 25%, SE = 6) and the most resistant strain (LF120, mean = 18%, SE = 6; paired t-test, t = 1.7, df = 3, P = 0.18).

For the susceptible strain, we also examined the effects of a 15:1 ratio of HaALP1f:Cry1Ac across a series of five concentrations of Cry1Ac activated toxin (Fig 8). The larval mortality was significantly higher for the mixture of HaALP1f and Cry1Ac than for Cry1Ac alone at each of the five concentrations of Cry1Ac (Fig 8). The LC_50_ (95% FL) of Cry1Ac was 29 (23.4 to 34.6) ng/cm^2^ without HaALP1f and 19 (13.3 to 22.7) ng/cm^2^, representing a significant, 1.6-fold synergy of Cry1Ac by HaALP1f.

## Discussion

The results here considered together with previous data indicate that several mechanisms are associated with resistance to Cry1Ac in lab- and field-selected populations of *H*. *armigera*, including reduced ALP activity and transcription, reduced APN activity, reduced conversion of protoxin to toxin, down-regulation of trypsin, mutations disrupting the extracellular and intracellular domains of cadherin, a mutation disrupting the ATP-binding cassette protein ABCC2, and mutations in unidentified genes [28–31, 57–65]. In the four most resistant strains of *H*. *armigera* studied here (LF10, LF30, LF60 and LF120), resistance to activated Cry1Ac toxin ranged from 48- to 1700-fold relative to a susceptible strain, and was associated with reduced ALP activity and transcription (Table 1, Figs 1 and 2).

The association between resistance to Cry1Ac and reduced ALP activity and transcription in these four strains is similar to results reported previously for resistant strains of three species of moths in the family Noctuidae, including the BtR strain of *H*. *armigera* [29]. The BtR strain was derived from the same susceptible strain we studied here (96S), selected in the laboratory with Cry1Ac protoxin in diet, and had >2900-fold resistance to Cry1Ac protoxin relative to 96S [29]. Relative to 96S, ALP activity was reduced by 2.3-fold in BtR [29] and 4.1-fold in LF120 (Fig 1); ALP transcription was reduced by 1.6-fold in BtR [29] and 2.9-fold in LF120 (Fig 2). In addition to reduced ALP activity, BtR also had reduced APN activity compared with 96S [29]. In contrast, previous data showed that expression of cadherin and APN was similar in LF120 and 96S [66]. Consistent with this previous result, APN activity did not vary significantly among the six strains analyzed here, including LF120 and 96S (Fig 3). However, we cannot exclude the possibility that several single amino acid differences found between LF120 and 96S in the cadherin and APN genes contribute to resistance [66]. The least resistant of the five selected strains studied here (LF5) had 22-fold resistance to Cry1Ac activated toxin and it did not differ from 96S in either ALP activity or transcription (Figs 1 and 2). Previous work identified *cis*-mediated down-regulation of trypsin as a mechanism of resistance in LF5 [64], mis-splicing of the ABCC2 gene as a mechanism of resistance in LF60 [65], and significantly reduced chemotrypsin-like activity in LF10 and LF30 relative to LF5 [63].

In a previous study, HaALP expressed in Sf9 cells bound activated Cry1Ac, but this binding depended on N-linked oligosaccharides [56]. Although proteins expressed in *E*. *coli* cells are usually not glycosylated, our two independent experiments using ligand blotting and SPR analysis confirmed that the HaALP1f expressed in *E*. *coli* bound activated Cry1Ac. Furthermore, at least three recent papers demonstrate binding of *E*. *coli*-expressed ALP to Cry toxins: binding of MsALP from *M*. *sexta* to Cry1Aa, Cry1Ab and Cry1Ac [67], binding of Aa-mALP from *Aedes aegypti* to Cry4Ba [68], and binding of a truncated ALP fragment (AgALP1t) from *Anopheles gambiae* to Cry11Ba [69]. The apparent affinity (K_d_) measured by SPR is 7.58 nM for HaALP1f-Cry1Ac binding in our study compared with 4 μM for MsALP-Cry1Ac binding reported for *Manduca sexta* [67]. This comparison shows that the binding affinity was 527-fold greater in our study.

The results here indicate that a fragment of ALP from *H*. *armigera* (HaALP1f) synergized Cry1Ac against a susceptible strain and all five resistant strains of *H*. *armigera* tested here. As far as we know, these results provide the first evidence that any ALP fragment synergizes a Bt toxin and the first evidence that any toxin-binding peptide synergizes a Bt toxin against a resistant strain. It remains to be determined if HaALP1f or other ALP fragments synergize Bt toxins against other strains of *H*. *armigera* or other pests. In future research aimed to determine the potential usefulness of toxin-binding fragments for managing resistance, it will be essential to test for synergism in resistant strains of pests.

## Materials and Methods

### Insect strains and rearing

We used six strains of *H*. *armigera*: one susceptible strain (96S) and five Cry1Ac-resistant strains (LF5, LF10, LF30, LF60 and LF120) [70]. The 96S strain was started with 20 pairs of adults collected from Xinxiang, Henan Province, China in 1996, and had been reared in the laboratory for >15 years on artificial diet without exposure to Bt toxins [71, 72]. The five resistant strains originated from the LF strain, which was started with 200 larvae collected from cotton fields in Langfang, Hebei Province, China in 1998 [73]. No permits were required because all collections were made in China under the auspices of the Chinese Ministry of Agriculture. Larvae from all strains were reared on diet. Rearing, selection, and bioassays were conducted at 27±2°C, photoperiod 14L:10D, and 75±10% relative humidity.

### Selection

We selected each of the five resistant strains with MVPII (Dow AgroSciences), a commercial formulation of CryAc protoxin [74–76] incorporated in diet at concentrations that yielded about 20% survival of neonates to the pupal stage [77]. First, we generated the LF5 strain by selecting the LF strain initially at 1 μg Cry1Ac protoxin per g diet for 38 generations (Table 2). In 2002, we increased the selection concentration for LF5 to 5 μg Cry1Ac protoxin per g diet. We maintained that concentration for 104 generations of selection for LF5. In 2003, we started the LF10 strain with a subset of LF5 and selected at 10 μg Cry1Ac protoxin per g diet. We used analogous methods to start the LF30, LF60, and LF120 strains (Table 2).

**Table 2:** Origins of Cry1Ac-resistant strains of *H*. *armigera.*

Strain	Parent strain	Year started	Selection concentration(μg per g diet)
LF5	LF*	2002	5
LF10	LF5	2003	10
LF30	LF10	2007	30
LF60	LF30	2008	60
LF120	LF60	2008	120

*Started in 1998 with 200 larvae collected from Langfang, Hebei Province, China.

### Bioassays

For each of the six strains of *H*. *armigera*, we used diet incorporation bioassays to determine the LC_50_ of Cry1Ac and diet overlay bioassays to determine the toxicity of Cry1Ac with and without HaALP1f. We used the activated form of Cry1Ac in all bioassays. To obtain activated Cry1Ac toxin, Cry1Ac protoxin was incubated 2 h at 37°C with a 25:1 ratio of trypsin (Sigma) to protoxin, and the soluble trypsinized toxin was purified by a Superdex 200 HR 10/30 column (Amersham Biosciences) on a fast protein liquid chromatography (FPLC) system. Cry1Ac protoxin was extracted and purified from the HD73 strain of *B*. *thuringiensis* subsp. *kurstaki* by the Biotechnology Group in Institute of Plant Protection, Chinese Academy of Agricultural Sciences, Beijing, China.

In all bioassays, we put one first instar in each well of a 24-well plate, with 24 first instars in each replicate and three replicates per treatment (total n = 72 per treatment). Larvae were considered dead if they died or did not reach third star after 7 days.

#### Diet incorporation bioassays to determine LC_50_ of Cry1Ac

We used diet incorporation bioassays to determine the LC_50_ of activated Cry1Ac toxin for each of the six strains. Various concentrations of Cry1Ac activated toxin were added and thoroughly mixed with diet to obtain the desired concentrations. After mixing, the diet solidified and we put pieces of solid diet (1 mg) into each well of a 24-well plate.

#### Diet overlay bioassays to determine effects of HaALP1f on toxicity of Cry1Ac

We used diet overlay bioassays to test the activated form of Cry1Ac with and without HaALP1f. Cry1Ac, purified HaALP1f, or both were diluted in PBS buffer, and incubated at 4°C for 1h. We poured 1.5 ml liquid diet into each well of a 24-well plate. After the diet solidified, 100 μl samples of the appropriate treatment materials (see below) were applied to the diet surface of each well and allowed to air dry.

Treatments consisted of activated Cry1Ac either alone (37 ng/cm^2^ for 96S, 99 ng/cm^2^ for LF5 and LF10, 620 ng/cm^2^ for LF30 and LF60, 1500 ng/cm^2^ for LF120) or with HaALP1f at four ratios of HaALP1f:Cry1Ac by weight (1:1, 5:1, 15:1 and 50:1). We also tested the susceptible 96S strain at a series of concentrations of Cry1Ac activated toxin (19 to 75 ng/cm^2^) either alone or in combination with HaALP1f at a fixed ratio of 15:1 HaALP1f:Cry1Ac by weight. We used three controls: untreated diet, diet treated only with PBS buffer, and diet treated only with HaALP1f in PBS buffer. In the treatments with only HaALP1f in PBS buffer, we tested the highest concentration of HaALP1f used in evaluating synergy for each strain, which ranged from 1870 ng/cm^2^ for 96S to 74,600 ng/cm^2^ for LF120.

### Brush border membrane vesicles (BBMV) and Aminopeptidase N (APN) Activity

We isolated BBMV by differential centrifugation [78], assessed by SDS-PAGE and kept them at -80°C until used. We measured BBMV protein concentration [79] using bovine serum albumin (BSA) (TransGene) as the standard.

The activity of APN, a marker enzyme for lepidopteran BBMV, was assayed by using leucine p-nitroanilide as the substrate [29]. For each strain, APN activity in the BBMV preparations was enriched six to eight-fold relative to the initial midgut homogenates. Three micrograms protein of each samples was used for assays. Enzymatic activities were monitored for 3 min as changes in optical density (OD) at 410 nm wavelength at room temperature in a microplate reader (BioTek). We calculated the maximum initial velocity (Vmax) using the Gen5 Data Analysis Software. For each of three independent preparations of BBMV, we measured APN activity three times for each of three samples in Oct. 2014.

### Alkaline phosphatase (ALP) activity

We determined ALP activity using a commercial kit (Alkaline phosphatase, Hou-Bio, P. R. China) as described by Jurat-Fuentes et al. (2011) [29]. Five μg protein of each samples was used for assays. Enzymatic activities were monitored for 2–3 min as changes in optical density (OD) at 405 nm wavelength at room temperature in a microplate reader (BioTek). We calculated the maximum initial velocity (Vmax) using the Gen5 Data Analysis Software. For each of three independent preparations of BBMV, we measured ALP activity three times for each of six samples.

### ALP RNA determination by quantitative real-time (qRT-PCR)

#### (1) RNA preparation and cDNA synthesis

Total RNA was extracted from midguts of 5th instar *H*. *armigera* larvae using Trizol reagent (Invitrogen) according to manufacturer’s instructions. Total RNA was treated with DNase I (TaKaRa) remove residual genomic DNA contamination. The integrity of total RNA was verified on a 1% agarose gel. Two μg RNA for each sample was reverse-transcribed with Quantscript RT Kit (TianGen, China) according to the manufacturer’s instructions. We used first strand cDNA as a template for qRT-PCR, with primers and reaction conditions as described previously for *H*. *armigera* [29].

#### (2) Primer design.

Oligonucleotide primers were designed using Primer 3 (http://frodo.wi.mit.edu/primer3/). Primers were made to amplify a 128 bp conserved region between the HaALP1 (accession no. EU729322) and HaALP2 (accession no. EU729323) isoforms. TaqMan probes (Invitrogen) were labeled at the 5’ end by the reporter dye FAM and at the 3’ end by the quencher dye TAMRA. Forward primer 5’ ATA GGC GTA GAC GGC ACG G 3’, reverse primer 5’ CGA GTC GTC GTC ACA ATA CCG 3’, and 5’-FAM CGC CGA GGA GAC TGT CAA GCC GCT T3’-TAMARA were used for HaALP fragment amplification. As endogenous control, we amplified a 184 bp fragment of *H*. *armigera* actin (accession no. X97615) with forward primer 5’ CAC AGA TCA TGT TCG AGA CGT TCA A 3’, reverse primer 5’- GCC AAG TCC AGA CGC AGG AT-3’ and 5’-FAM CCG CCA TGT ACG TCG CCA TCC AGG 3’-TAMARA.

#### (3) Real-time PCR reactions and data analysis

We performed qRT-PCR in triplicate for each of at least three independent biological samples using methods similar to Jurat-Fuentes et al. 2011 [29]. Reactions for each *H*. *armigera* sample (25 μl) consisted of 12.5 μl of Premix *Ex* Taq (2×) (TaKaRa), 0.5 μl of Rox Reference DyeII (50×), probe (0.2 μM), primers (0.4 μM), 1 μl of sample cDNA and 8.5μl sterilized ultrapure water.

Amplification conditions were an initial denaturation at 95°C for 2 min followed by 40 cycles of 95°C for 15 s, and a single step for annealing and extension was done at 60°C for 60 s. We used a relative quantitation method (2^-ΔΔCT^) [80] to evaluate quantitative variation. Transcript amounts were standardized to 1 with the sample from susceptible larvae containing the highest transcript levels from the three biological replicate reactions performed.

### Cloning, expression and purification of HaALP1f

A 780 bp cDNA fragment of the gene encoding HaALP1 [56] was cloned and expressed in *E*. *coli* as a His-tag recombinant protein. Total RNA was extracted and treated with DNase I (TaKaRa) as described above, then reverse-transcribed with SuperScript III RNase H^-^ reverse-transcriptase (Invitrogen). The cDNA fragments were used as a template for PCR amplification using primers ALP-F (5’-CGGGATCCGCGAAGACGGCGAACCGCACCTG-3’ BamHI) and ALP-R (5’-CGCTCGAGAGTGCGATAGTTTGGCTCAAGGGT-3’ XhoI). The PCR products were purified with DNA purification system Kit (Biomed) and cloned into the pMD 19-T simple vector (TaKaRa) following the manufacturer’s instructions. The recombinant plasmid was excised with BamHI and XhoI, subcloned into the His-tagged expression vector pET28a+ (Novagen), and transfected into *E*. *coli* BL21 (DE3) cells (Transgen, China). The transformants were cultured overnight with constant agitation at 37°C in 5 ml of Luria-Bertani (tryptone 1.0% (w/v), yeast extract 0.5% (w/v) and NaCl 1.0% (w/v), supplemented with 20 mg/L Kanamycin). The following morning, 4 ml of this overnight culture were used to inoculate 400 ml of LB broth plus 20 mg/L kanamycin in a 1000 ml flask. This culture was allowed to grow at 37°C with constant agitation until reaching an OD of 0.7 at 600_nm_, and expression was induced with 0.2 mM Isopropyl β-D-1-thiogalactopyranoside (IPTG) for 5 h at 37°C with constant agitation.

For purification of the expressed fragment (HaALP1f), pellets of cultured *E*. *coli* BL21 cells transformed with the ALP fragment-pET28a+ construct were collected by centrifugation at 3000×g, 4°C for 20 min, washed with 50 ml of ice-cold PBS buffer, re-suspended in 30 ml of PBS buffer, and sonicated for 15 min on ice. After 25,000×g centrifugation for 20 min at 4°C, the expressed HaALP1f fragment as inclusion bodies were solubilized with 15 ml of 8M urea in PBS buffer. The solubilized fragments were subjected to affinity purification using Ni-Sepharose beads (Amersham Biosciences). The HaALP1f fragments were eluted with 15 ml of 500 mM imidazole and dialyzed against PBS buffer. The purified ALP fragments were separated by 10% SDS-PAGE.

### Detection of HaALP1f and binding of Cry1Ac to HaALP1f

Western blot analysis was used to detect expression of HaALP1f. Purified HaALP1f was separated in a 10% SDS-PAGE gel and transferred to a polyvinylidene difluoride (PVDF) membrane fllter. At room temperature, the PVDF membrane was blocked for 2 h in 25 ml of blocking buffer (PBS buffer, 5% skim milk powder, pH 7.4), hybridized with 2.5 μl of anti-His-tag monoclonal antibody (Cal Bio, China) (1:10,000) for 1 h, and washed five times (5 min each) with 25 ml of PBST (PBS, 0.1% Tween-20, pH 7.4). After washing, the PVDF membrane was probed with 1.5 μl of HRP-conjugated secondary antibody (ZSGB-BIO, China) (1:20,000) for 1 h at room temperature. The resultant his-tagged HaALP1f fragment peptide-antibody complex on the PVDF membrane was visualized using the Super ECL Plus Detection Kit (Applygen, China).

We used ligand blot analysis to detect binding of Cry1Ac to purified HaALP1f. As in Western blot analysis, this analysis was performed at room temperature and purified HaALP1f was separated in a 10% SDS-PAGE gel and transferred to a PVDF membrane. The PVDF membrane was blocked for 2 h with 25 ml blocking buffer (PBS buffer, 5% skim milk powder, pH 7.4), incubated in 25 ml of PBST buffer (PBS buffer, 0.1% Tween-20, pH 7.4) containing 3 μg of activated Cry1Ac toxin for 1.5 h, and washed. After washing five times (as described above), the PVDF membrane was incubated with 2.5 μl of polyclonal anti-Cry1Ac antibody (1:10,000) for 1.5 h at room temperature in PBST buffer. After washing again, the PVDF membrane was probed with 1.5 μl of an HRP-conjugated secondary antibody (1:20,000) for 1 h, and visualized as described above.

SPR experiment was performed on a BIAcore3000 machine (Biocore AB). Cry1Ac toxin in 10mM sodium acetate, pH4.0, was immobilized on a CM5 sensor chip by amine coupling method (Biacore AB). The flow buffer HBS (10 Mm HEPES, 150mM NaCl, 0.005% Tween20(v/v), pH7.4) was used at a flow rate of 40μl/min. Multiple concentrations (31.25nM, 62.5nM, 125 nM, 250 nM, 500 nM) of HaALP1f was injected across the flow cell containing the Cry1Ac and one blank flow cell containing ethanolamine as a blocking agent. Surfaces were regenerated with a 30-second injection of 5 mM NaOH at a flow rate of 40μl/min. Signal responses from the blank flow cells were subtracted from all response curves and data were locally fitted using BIAevaluation Ver. 4.1 (Biacore AB). The curves were fitted to a simple 1:1 Langmuir binding model (A+B ↔AB) to obtain apparent rate constants.

### Statistical analyses

We used POLO [81] to estimate the concentration of Cry1Ac activated toxin killing 50% (LC_50_) and its 95% fiducial limits, as well as the slope of the concentration-mortality line and its standard error (SE). We calculated resistance ratio as the LC_50_ of a strain divided by the LC_50_ of the susceptible strain (96S).

We used analysis of variance (ANOVA) with Duncan’s multiple range test for multiple comparisons (P < 0.05) to evaluate a) variation among strains in relative ALP transcription; b) variation within strains in mortality among each of the eight controls and treatments in diet overlay bioassays; and c) variation among strains in ALP activity and transcription, and d) variation among strains in APN activity. We used linear regression to assess the relationship between ALP activity and both resistance ratio (log-transformed) and ALP transcription [82].

We tested for synergism using what Tabashnik [83] described as “perhaps the simplest approach,” by comparing mortality caused by Cry1Ac alone with mortality caused by sublethal concentrations of HaALPf1. The controls with only HaALPf1 and PBS buffer showed that HaALPf1 at the highest concentrations tested in combination with Cry1Ac did not cause mortality (Results). In this case, assuming no synergism, adding HaALPf1 to Cry1Ac is not expected to increase mortality. Thus, significantly greater mortality caused by combinations of Cry1Ac and HaALPf1 relative to Cry1Ac alone indicates synergism [83]. This approach has been applied previously to evaluate synergism between Cry toxins [84], between Cry and Cyt toxins [85], and between Cry toxins and fragments of cadherin-binding proteins [46]. For each of the four ratios of HaALPf1 to Cry1Ac (1:1, 5:1, 15:1 and 50:1) tested against each strain, we used ANOVA with Duncan’s multiple range test for multiple comparisons (P < 0.05) to determine if the mean mortality with the combination of Cry1Ac and HaALPf1 was significantly greater than the mean mortality with Cry1Ac alone (Fig 7). For the susceptible 96S strain, we also examined the effects of a 15:1 ratio of HaALP1f:Cry1Ac across a series of five concentrations of Cry1Ac activated toxin (Fig 8). In addition to comparing mortality of the combination of Cry1Ac and HaALP1f versus Cry1Ac alone at each concentration as described above, we used POLO [81] to estimate the LC_50_ of Cry1Ac with and without HaALP1f. We used these data to test for synergism by determining if the LC_50_ with HaALP1f was significantly greater than the LC_50_ without HaALP1f, as indicated by non-overlap of the 95% fiducial limits.

## Supporting Information

S1 Table Data for Figs 1 and 3 APN & ALP activity. (XLSX)

S2 Table Data for Fig 2 ALP transcription. (XLS)

S3 Table Data for Fig 6 Cry1Ac binding to HaALP1f. (XLSX)

S4 Table Data for Fig 7 Synergism of Cry1Ac by HaALP1f vs. susceptible & resistant strains. (XLS)

S5 Table Data for Fig 8 Synergism of Cry1Ac by HaALP1f vs. susceptible strain. (XLSX)

S6 Table Data for enrichment of APN activity in BBMV. (XLS)
